# Efficacy and Mechanism of Synbiotics in Relieving Functional Constipation: Optimized by Generation Time

**DOI:** 10.3390/nu18020184

**Published:** 2026-01-06

**Authors:** Linlin Wang, Huahao Cai, Qingwei Yao, Zehua Chen, Wenzhi Li, Cencen Liu, Shumao Cui

**Affiliations:** 1 State Key Laboratory of Food Science and Resources, Jiangnan University, Wuxi 214122, China; wanglinlin@jiangnan.edu.cn (L.W.); huahao.cai@teshiyuan.com (H.C.); 2 School of Food Science and Technology, Jiangnan University, Wuxi 214122, China; 3 Wuxi Institute for Specialized Nutrition and Health Co., Ltd., Wuxi 214142, China; qw.yao@teshiyuan.com; 4 Infinitus (China) Company Ltd., Guangzhou 510080, China; jeff.chen@infinitus-int.com (Z.C.); peter.wenzhi.li@infinitus-int.com (W.L.)

**Keywords:** probiotics, prebiotics, generation time, synergy, constipation

## Abstract

**Background:** Functional constipation (FC) represents a highly prevalent gastrointestinal disorder, affecting approximately 8.5% of the population in China. It is frequently associated with anxiety and depression, significantly impairing patients’ quality of life. Conventional microecological therapeutic approaches predominantly rely on empirical probiotic–prebiotic combinations. However, these pairings are seldom selected based on strain-specific metabolic characteristics, ultimately leading to suboptimal therapeutic synergy. **Methods:** The generation time (GT) of four constipation-relief strains was measured across eight oligosaccharides to identify optimal substrates for synbiotic formulation. The GT-optimized synbiotic was verified in a loperamide-induced mouse model vs. single probiotics/prebiotics. The related mechanisms of were assessed through 16S rDNA sequencing, targeted metabolomics, and qPCR. **Results:** The GT-optimized synbiotic significantly outperformed all single components. Specifically, the synbiotic significantly decreased the time to first black stool and increased fecal water content. Mechanistically, it restored colonic neurotransmitter balance, suppressed aquaporin expression, enriched butyrate-producing bacteria, and repaired barrier integrity. Overall, these effects work together, increasing the moisture content of the feces and accelerating intestinal peristalsis, ultimately alleviating constipation. **Conclusions:** We propose a GT-guided precision-pairing strategy that identifies optimal prebiotics based on strain-specific generation times, demonstrating synergistic enhancement of short-chain fatty acid (SCFA) production, enteric neurotransmitter signaling, and aquaporin-mediated water transport. This GT guided synbiotic approach shows promise in preclinical models and warrants validation in human trials.

## 1. Introduction

Functional constipation (FC) represents a highly prevalent gastrointestinal motility disorder. Epidemiological studies indicate that the overall prevalence of FC is approximately 8.5%, with a sustained upward trend observed from 1991 to 2020 [1]. FC imposes a substantial burden on public quality of life. It can lead to serious anorectal complications, such as pain and hemorrhoids, and is significantly associated with psychological comorbidities, including anxiety, depression, and impaired sleep quality [2].

The pathophysiology of functional constipation (FC) involves multifactorial mechanisms. Key contributing factors include impaired colonic motility, disturbances in neurotransmitter secretion, dysfunction of the intestinal epithelial barrier, and gut microbiota dysbiosis [3]. Crucially, gut microbiota dysbiosis can further impair epithelial barrier integrity and disrupt neurotransmitter signaling. This disruption leads to uncoordinated intestinal peristalsis, which deteriorates the gut microenvironment. Such alterations adversely affect the gut microbial community, creating a vicious cycle that exacerbates constipation symptoms.

While traditional pharmacotherapies (such as stimulant or osmotic laxatives) offer rapid symptom relief, prolonged use may lead to adverse effects such as dependence and electrolyte imbalance, limiting long-term utility, highlighting the urgent need for safer therapeutic alternatives [4].

Microecological interventions (including probiotics and/or prebiotics) have garnered significant attention due to their capacity to restore microbial homeostasis [5]. Probiotics reinforce the epithelial barrier, stimulate short-chain fatty acid (SCFA) production, and modulate enteric neurotransmitter activity, while prebiotics, as indigestible dietary components, selectively promote the proliferation of beneficial bacteria [6,7,8,9].

Synbiotics confer prebiotic and probiotic benefits and their synergistic effects, potentially ensuring an increase in SCFAs, improving tight junctions and mucin production, lowering the intestinal pH, and balancing gut microbiota composition [10]. However, synbiotics are the least investigated substances regarding health effects, compared to prebiotics and probiotics, and their mechanisms still need to be better understood [11].

Most published studies have focused on evaluating the efficacy of single probiotics or prebiotics, with systematic strategies for selecting multi-strain–multi-substrate combinations remaining scarce [12]. Prebiotic selection is typically guided by empirical rather than quantitative criteria, thereby limiting the full exploitation of probiotic–prebiotic interactions. For instance, the impact of individual oligosaccharides on the generation time (GT) of each probiotic within a mixed consortium has not been systematically assessed, precluding precise alignment of carbon sources with strain-specific metabolic demands.

Prebiotics primarily function as trophic substrates that support probiotic proliferation and cellular growth. The ability of probiotics to utilize prebiotics depends on several critical metabolic pathways, including carbon catabolite repression mechanisms that regulate monosaccharide preference [13], as well as the presence of specialized carbohydrate-active gene clusters in the genome. These encompass carbohydrate transport systems, glycosyl hydrolases, and phosphorylases enzymatic tools dedicated to the breakdown of non-digestible oligosaccharides [14]. The repertoire of carbohydrate metabolism genes possessed by a probiotic strain is a key determinant of its GT [15]. Consequently, measuring the GT during probiotic utilization of prebiotics offers a promising quantitative approach for screening and optimizing probiotic–prebiotic pairings in synbiotic formulations, addressing a significant gap in current microecological therapeutic design.

This study hypothesizes that the prebiotic combination selected through the “shortest-GT oligosaccharide prioritization” strategy enhances carbon utilization efficiency in the targeted strains (*Bifidobacterium longum* CCFM1167, *B. animalis* subsp. *lactis* CCFM1278, *B. longum* subsp. *longum* CCFM1113, *Pediococcus acidilactici* CCFM6432), thereby promoting microbial colonization and functional expression. The alleviation of constipation observed in the model was hypothesized to involve multi-pathway mechanisms, involving enteric nervous system modulation, intestinal barrier repair, and upregulation of aquaporin expression.

Accordingly, the present study was designed to (i) evaluate eight oligosaccharides (isomaltulose, resistant dextrin, stachyose, xylo-oligosaccharide, lactulose, polydextrose, soybean oligosaccharide, and inulin) for their capacity to minimize the GT of each target strain; (ii) develop a GT-optimized synbiotic formulation; and (iii) validate its efficacy in a loperamide-induced mouse model of functional constipation (FC) while elucidating the underlying mechanisms through multi-omics analyses.

## 2. Materials and Methods

### 2.1. Materials and Reagents

Loperamide hydrochloride capsules were purchased from Xi’an Janssen Pharmaceutical Ltd. (Xi’an, China). Gum Arabic and activated charcoal powders were procured from Sinopharm Chemical Reagent Co., Ltd. (Shanghai, China). ELISA kits for quantifying mouse acetylcholine (ACh), substance P (SP), vasoactive intestinal peptide (VIP), and inducible nitric-oxide synthase (iNOS) were provided by Shanghai Enzyme-linked Biotechnology Co., Ltd. (Shanghai, China).

The activated-charcoal suspension was prepared by dissolving 10 g of gum Arabic in 80 mL of distilled water with heating until complete dissolution. Subsequently, 5 g of activated charcoal was added, and the mixture was boiled three times. After cooling to room temperature, the volume was adjusted to 100 mL with distilled water. The suspension was stored at 4 °C and vigorously shaken before each use.

### 2.2. Prebiotic Screening for Optimal Composition

The prebiotic selection protocol was adapted from Chen et al. [16]. Strains revived through 2–3 passages were inoculated at 2% (*v*/*v*) into modified MRS broth supplemented with a test oligosaccharide as the sole carbon source. Cultures were incubated at 37 °C under anaerobic conditions. MRS broth with glucose served as the positive control, while sterile medium without inoculation acted as the blank control. Optical density at 600 nm (OD_600_) was monitored spectrophotometrically. Two time points within the exponential growth phase were selected for GT calculation using Equation (1):

(1) $$GT=\frac{t_{2}-t_{1}}{3.322\times (A_{2}-A_{1})}$$

where *t*_2_ and *t*_1_ are the time points (h), and *A*_2_ and *A*_1_ are the corresponding OD_600_ values.

### 2.3. Animal Experimental Design

The four probiotic strains involved in this animal experiment were all sourced from the Culture Collection of the Food Microorganisms Center, Jiangnan University. Detailed information is shown in Table 1.

Activated strains were inoculated at 2% (*v*/*v*) into MRS liquid medium supplemented with 0.5% L-cysteine hydrochloride and cultured anaerobically at 37 °C for 18 h. The resulting bacterial cells were harvested by centrifugation and resuspended in a 30% sterile glycerol solution. The bacterial suspensions were stored at −80 °C for subsequent oral gavage administration. Prior to administration, the frozen bacterial suspensions were rapidly thawed in a 37 °C water bath and centrifuged. The pellet was washed with physiological saline to remove the cryoprotectant. Finally, the bacteria were resuspended and diluted in physiological saline to the desired concentration for gavage.

All animal procedures utilized 8-week-old male C57BL/6J mice procured from Beijing Vital River Laboratory Animal Technology Co., Ltd., Beijing, China. Animals were maintained in a specific pathogen-free (SPF) facility under controlled environmental parameters: temperature (23 ± 2) °C and relative humidity (50 ± 10)%, with a standardized 12 h light/dark cycle. The mice were provided unrestricted access to water and a chow diet (XTI01CR-010, Jiangsu Province Cooperative Pharmaceutical Bioengineering Co., Ltd., Nanjing, China). The study protocol received approval from the Institutional Animal Care and Use Committee (IACUC-JIPD-2024-102) and was performed in accordance with EU Directive 2010/63/EU [17].

**Figure 1:**
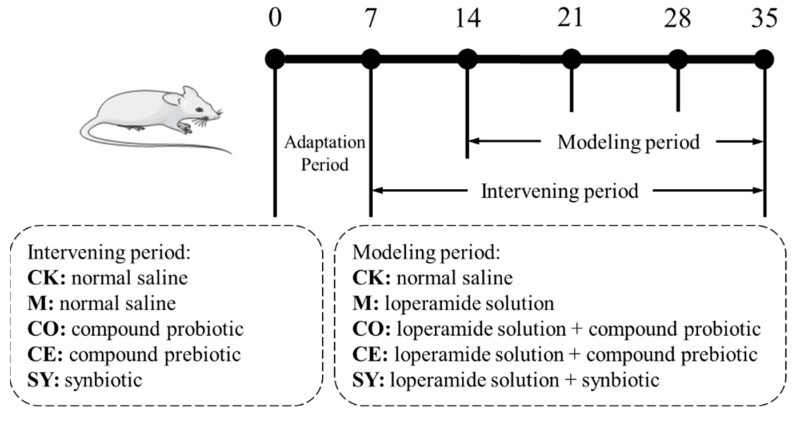
Experimental protocol for animal study on functional constipation.

The animal experiment was conducted following a protocol adapted from Zhang et al. [18] and Shen et al. [19]. The experimental groups and procedures are presented in Figure 1 and Table 2. Following a 7-day acclimatization period, 30 mice were randomly allocated into six experimental groups (6 mice per group): (1) Control check group (CK), (2) Model group (M), (3) Compound probiotic group (CO), (4) Compound prebiotic group (CE), and (5) Synbiotic (SY) group.

Throughout the intervening period, all groups received respective interventions: CK and M groups—sterile saline; CO group—bacterial suspension containing four probiotic strains (*Bifidobacterium longum* CCFM1167, *B. animalis* subsp. *lactis* CCFM1278, *B. longum* subsp. *longum* CCFM1113, *Pediococcus acidilactici* CCFM6432) at 1 × 10^9^ CFU/mL in 0.2 mL sterile saline; CE group—0.22 g/mL prebiotic mixture (0.2 mL); SY group—combined synbiotic formulation (0.2 mL). The doses for probiotics [20,21,22,23] and prebiotics [24,25,26] were selected based on previous studies.

During the modeling period, the constipation model was established by administering loperamide hydrochloride at a dose of 10 mg/kg body weight everyday [18]. In detail, one hour prior to the interventions, the constipation model was established by oral administration of 0.2 mL loperamide hydrochloride (10 mg/kg body weight), while the CK group received equivalent volumes of sterile saline.

### 2.4. Fecal Water Content

Mice were placed individually in clean cages lined with filter paper. All fresh feces excreted within a fixed period were collected into pre-weighed sterile tubes and weighed to obtain the wet mass. Samples were then lyophilized to constant weight using a vacuum freeze-dryer. Fecal water content was calculated with Equation (2):

(2) $$Fecal\;water\;content\left(\%\right)=\frac{wet\;weight(g)-dry\;weight(g)}{wet\;weight(g)}\times 100\%$$

### 2.5. Time to First Black Stool

Gastrointestinal transit time was evaluated in line with a previous research method [18]. On the day before sacrifice, CK mice received 0.2 mL of saline, whereas all other groups were dosed with loperamide (10 mg kg^−1^ BW, 0.2 mL). One hour later, the CK and M groups received 0.2 mL of saline plus charcoal ink, while intervention groups received 0.2 mL of bacterial suspension (or synbiotic) plus charcoal ink. Each animal was transferred to a clean cage with absorbent paper; the interval between ink administration and the appearance of the first black stool was recorded as the time to first black stool.

### 2.6. Small-Intestinal Transit Rate

A laparotomy was performed, and the intestinal segment from the pylorus to the ileo-cecal valve was excised. The excised segment was then laid out in a straight configuration without tension and photographed. Measurements included the total length of the small intestine and the distance covered by the charcoal front from the pylorus. Transit rate was derived using Equation (3):

(3) $$Transit\;rate(\%)=\frac{charcoal\;front\;distance(cm)}{total\;intestinal\;length(cm)}\times 100\%$$

### 2.7. Colonic mRNA Quantification

A 5 mm mid-colon segment was snap-frozen in liquid nitrogen and stored at −80 °C. Total RNA was extracted with a commercial kit, reverse-transcribed to cDNA, and analyzed by RT-qPCR using SYBR Green master mix (Q712, Nanjing Vazyme Biotech Co., Ltd., Nanjing, China). Gapdh (Glyceraldehyde-3-phosphate dehydrogenase) served as the housekeeping gene. Transcripts related to enteric neurotransmitters, mechanical barrier, immune barrier, gut-regulatory peptides, and aquaporins were quantified. Primer sequences are listed in Table 3.

### 2.8. Histopathological Examination

Mid-colon segments were fixed overnight in 4% paraformaldehyde, rinsed, dehydrated through graded ethanol, cleared in xylene, and embedded in paraffin. Sections of 4 µm were stained with hematoxylin and eosin (H&E) and scanned with a digital slide scanner for morphometric evaluation.

### 2.9. Quantification of Fecal Short-Chain Fatty Acids

Fecal SCFA levels were measured using gas chromatography–mass spectrometry (GC-MS) based on the method reported by Wang et al. [27]. The procedure involved suspending 30 mg of lyophilized feces in 0.5 mL of saturated NaCl solution, homogenizing the suspension, and acidifying it with 20 µL of 10% H_2_SO_4_, followed by vortex mixing for 30 s. Subsequently, 1 mL of diethyl ether was added, and the mixture was centrifuged at 12,000× *g* for 15 min at 4 °C. The ether phase was then transferred, dried over 0.3 g of anhydrous Na_2_SO_4_, re-centrifuged, and 1 µL of the supernatant was injected into a Rtx-Wax column (30 m × 0.25 mm × 0.25 µm) on a GC-MS system. Helium carrier gas flow was maintained at 2 mL min^−1^. The oven temperature program included an initial hold at 20 °C, a ramp at 7.5 °C min^−1^ to 140 °C, and a final hold for 3 min at 200 °C. Full-scan mode was applied, and an external calibration curve was utilized for quantification.

### 2.10. 16S rDNA Amplicon Sequencing and Bioinformatics

Bacterial DNA was extracted from 200 mg of feces using a Fecal FastDNA Spin Kit (6570200, MP Biomedicals LLC, Solon, OH, USA). The V3–V4 region was amplified with primers 341F/806R, and amplicons were verified on 1.5% agarose containing 4% nucleic-acid stain, purified with the DNA Gel/PCR Purification Miniprep Kit (BW-DC3510-01, BIOMIGA Inc., San Diego, CA, USA), and quantified on a Nanodrop spectrophotometer (2000C, Thermo Scientific, Waltham, MA, USA). Equimolar pools were prepared and sequenced on an Illumina MiSeq platform with a MiSeq Reagent Kit v3 (2 × 300 bp, Illumina, Inc., San Diego, CA, USA). Raw reads were demultiplexed and quality-filtered in QIIME2 (v2022.8). ASVs were resolved with DADA2, taxonomy assigned against the SILVA 138 database, and downstream analyses (α-diversity, β-diversity, LEfSe) performed using the QIIME2 plugins.

### 2.11. Data Analysis

All data are expressed as mean ± SD. Differences between the normal control (CK) and the constipation model (M) were evaluated by Student’s unpaired two-tailed *t*-test; # *p* < 0.05, ## *p* < 0.01, ### *p* < 0.001. Comparisons among M and the intervention groups were performed by one-way ANOVA followed by Duncan’s post hoc test versus M; * *p* < 0.05, ** *p* < 0.01, *** *p* < 0.001. Statistical analyses were conducted with SPSS 26.0, and graphs were prepared using GraphPad Prism 9.

## 3. Results

### 3.1. Screening of Probiotic–Prebiotic Synbiotic Formulations

**Figure 2:**
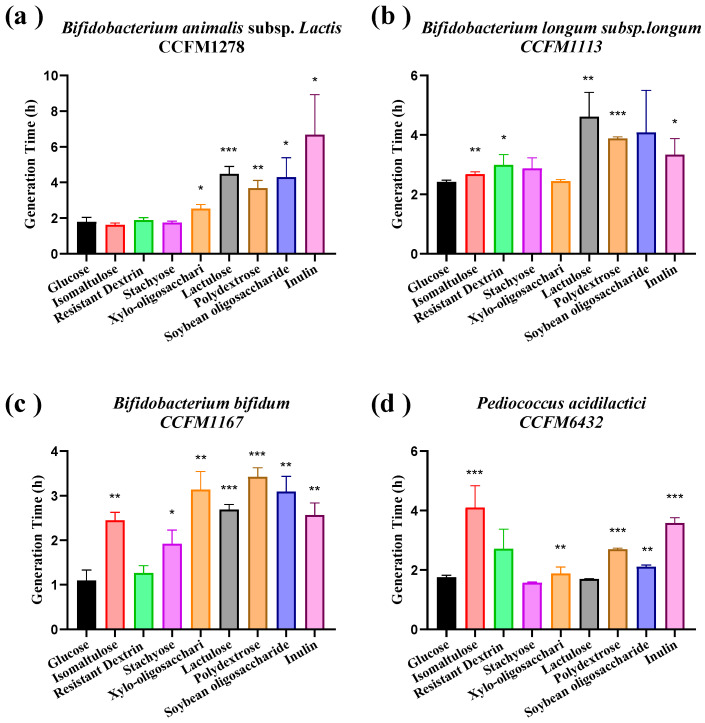
Generation time of probiotics grown on different oligosaccharides as the sole carbon source. (**a**) *B. animalis* subsp. *lactis* CCFM1278, (**b**) *B. longum* subsp. *longum* CCFM1113, (**c**) *B. longum* CCFM1167, and (**d**) *Pediococcus acidilactici* CCFM6432. Data represent mean ± SEM. Significance levels (* *p* < 0.05, ** *p* < 0.01, *** *p* < 0.001 compared to the glucose-grown controls).

The GT of four strains (*Bifidobacterium longum* CCFM1167, *B. animalis* subsp. *lactis* CCFM1278, *B. longum* subsp. *longum* CCFM1113, and *Pediococcus acidilactici* CCFM6432) was determined in modified MRS containing each of eight oligosaccharides as the sole carbon source. No significant difference in GT versus glucose-grown controls (*p* > 0.05) was observed for *B. animalis* subsp. *lactis* CCFM1278 on isomaltulose, resistant dextrin, or stachyose; *B. longum* subsp. *longum* CCFM1113 on isomaltulose or xylo-oligosaccharide; *B. longum* CCFM1167 on resistant dextrin; and *P. acidilactici* CCFM6432 on resistant dextrin or stachyose. Consequently, the optimal synbiotic combination was defined as the four strains paired with isomaltulose, xylo-oligosaccharide, resistant dextrin, and stachyose (Figure 2).

### 3.2. Evaluation of the Efficacy of Probiotic–Prebiotic Compound in Alleviating Constipation

**Figure 3:**
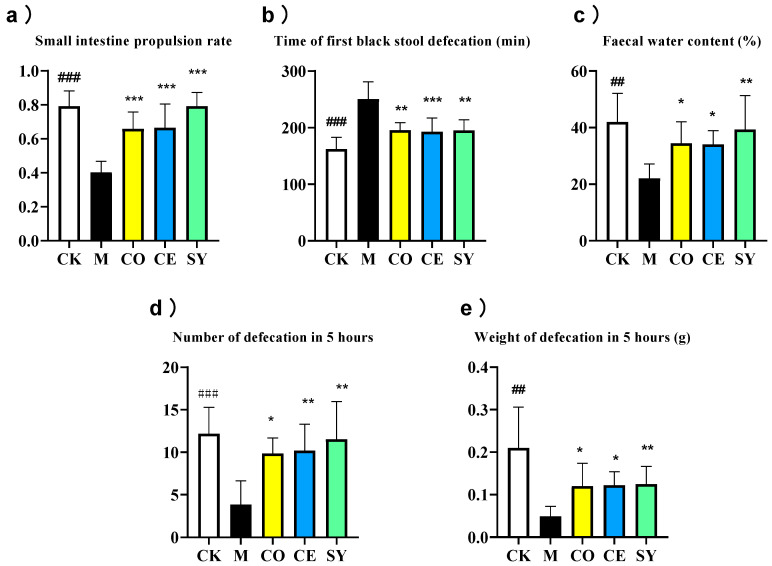
Effect of different interventions on constipation-related phenotypic indices in constipated mice. (**a**) Small-intestinal propulsion rate, (**b**) time of first black stool defecation, (**c**) fecal water content, (**d**) number of defecations in 5 h, and (**e**) weight of defecation in 5 h. Data represent mean ± SD. Significance levels (## *p* < 0.01, ### *p* < 0.001 compared to CK group; * *p* < 0.05, ** *p* < 0.01, *** *p* < 0.001 compared to M group).

Loperamide activates μ-opioid receptors, triggering Gi/o-coupled signaling and arrestin-mediated MAPK phosphorylation, which suppresses smooth-muscle contraction and slows transit, thereby inducing functional constipation [28]. Compared with the normal control group, the model group showed significant decreases in small-intestinal propulsion rate, fecal water content, and the number and weight of fecal pellets within 5 h (Figure 3, *p* < 0.001), while the time to first black stool excretion was significantly prolonged (Figure 3, *p* < 0.001), confirming successful model construction. Intervention results demonstrated that the SY group exhibited the most pronounced therapeutic effects, with a 96.4% increase in small-intestinal propulsion rate, a 22.1% reduction in time to first black stool excretion, a 78.2% increase in fecal water content, and 200.0% and 154.4% increases in the number and weight of fecal pellets within 5 h, respectively (Figure 3, *p* < 0.01). The CO and CE groups also significantly improved these parameters (small-intestinal propulsion rates increased by 63.1% and 64.7%, respectively; time to first black stool excretion decreased by 22.0% and 23.1%, respectively; fecal water content increased by 56.1% and 54.6%, respectively; 5 h fecal pellet counts increased by 156.5% and 165.5%, respectively; fecal weights increased by 144.6% and 149.3%, respectively), but their effects were inferior to those of the SY group (Figure 3, *p* < 0.05). Comprehensive analysis indicated that the SY group outperformed the CO and CE groups in improving small-intestinal propulsion rate, fecal water content, and 5 h fecal pellet count.

Given the SY group’s significant efficacy in the constipation model, subsequent studies focused on elucidating the mechanism, with a particular emphasis on four dimensions: regulation of gastrointestinal neurotransmitter secretion, expression of proteins related to intestinal water absorption and secretion, intestinal microecological balance, and intestinal immune modulation.

### 3.3. Evaluation of the Efficacy of Probiotic–Prebiotic Compound in Gastrointestinal Regulatory Neurotransmitters in Constipated Mice

**Figure 4:**
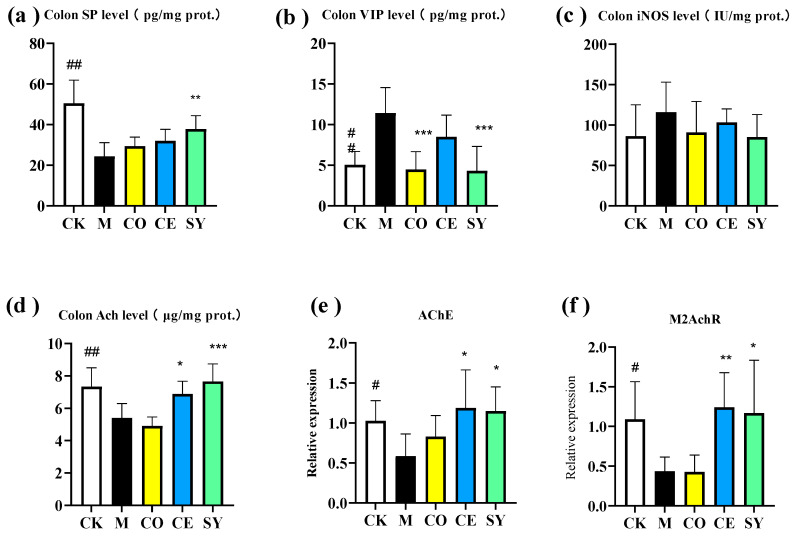
Effect of different interventions on gut-regulatory neurotransmitters in constipated mice. The colon levels of (**a**) SP, (**b**) VIP, (**c**) iNOS, and (**d**) Ach, and the relative mRNA expression of (**e**) *AChE* and (**f**) *M2AChR*. Data represent mean ± SD. Significance levels (# *p* < 0.05, ## *p* < 0.01, compared to CK group; * *p* < 0.05, ** *p* < 0.01, *** *p* < 0.001 compared to M group).

This study measured the levels of multiple gastrointestinal regulatory transmitters in the colons of constipated mice, including the excitatory regulatory transmitter substance P (SP), the inhibitory regulatory transmitters vasoactive intestinal peptide (VIP) and nitric oxide (NO), and the neurotransmitter acetylcholine (ACh) and its receptor (muscarinic acetylcholine receptor, M2AChR), as well as acetylcholinesterase (AChE) activity. The results showed that in the model group, the protein levels of SP and ACh in the colon, as well as the mRNA expression of AChE and M2AChR, were significantly decreased (Figure 4, *p* < 0.05), while the protein level of VIP was significantly increased (Figure 4, *p* < 0.01) and iNOS activity was enhanced. The intervention results revealed that compared to the model group, the SY group exhibited significantly upregulated protein levels of SP and ACh and mRNA expression of AChE and M2AChR in the colon (Figure 4, *p* < 0.05) and a significantly downregulated protein level of VIP (Figure 4, *p* < 0.001). The CO group only showed a significantly downregulated VIP content (Figure 4, *p* < 0.001), and the CE group demonstrated significantly upregulated levels of ACh, AChE, and M2AChR (Figure 4, *p* < 0.05).

Comprehensive analysis indicated that prebiotics and probiotics exhibited complementary and synergistic effects in regulating colonic neurotransmitter and receptor expression: they could independently enhance AChE and M2AChR mRNA expression (complementary effect) and jointly increase SP and ACh levels while reducing VIP levels (synergistic effect), thereby more comprehensively regulating the balance between excitatory and inhibitory transmitters and enhancing the function of the cholinergic signaling pathway. This further validated that the combined intervention of probiotics and prebiotics achieved superior constipation relief by multi-targeted regulating of gastrointestinal regulatory transmitters.

### 3.4. Evaluation of the Efficacy of Probiotic–Prebiotic Compound in Intestinal Aquaporin in Constipated Mice

**Figure 5:**
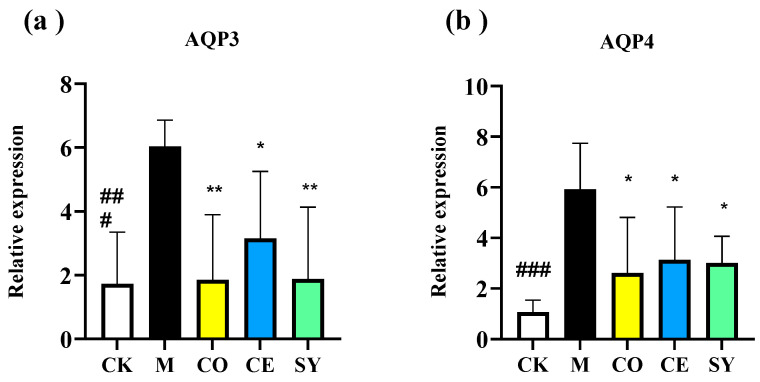
Effect of different interventions on intestinal water absorption channels in constipated mice. The relative mRNA expression of (**a**) AQP3 and (**b**) AQP4. Data represent mean ± SD. Significance levels (### *p* < 0.001 compared to CK group; * *p* < 0.05, ** *p* < 0.01 compared to M group).

The mRNA expression levels of AQP3 and AQP4 in the mouse colon were significantly increased in the model group compared to the CK group (Figure 5, *p* < 0.001). However, all three intervention groups (CO, CE, and SY) significantly downregulated the expression of AQP3 and AQP4 (Figure 5, *p* < 0.05), indicating no significant differences among the three groups in improving intestinal water absorption and secretion.

### 3.5. Evaluation of the Efficacy of Probiotic–Prebiotic Compound in Gut Microbiota in Constipated Mice

**Figure 6:**
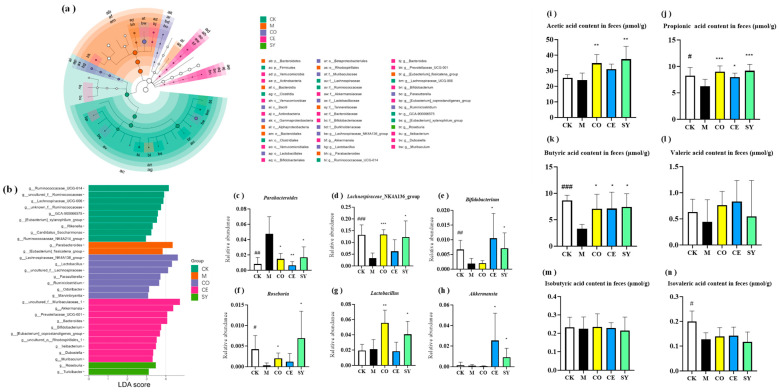
Effect of different interventions on gut microbiota in constipated mice. (**a**) LEfSe cladogram and (**b**) LEfSe bar. The relative abundances of (**c**) *Parabacteroides*, (**d**) *Lachnospiraceae_NK4A136_group*, (**e**) *Bifidobacterium*, (**f**) *Roseburia*, (**g**) *Lactobacillus*, and (**h**) *Akkermansia*. SCFA contents in feces: (**i**) acetic acid, (**j**) propionic acid, (**k**) butyrate acid, (**l**) valeric acid, (**m**) isovaleric acid, and (**n**) isobutyric acid. Data represent mean ± SD. Significance levels (# *p* < 0.05, ## *p* < 0.01, ### *p* < 0.001 compared to CK group; * *p* < 0.05, ** *p* < 0.01, *** *p* < 0.001 compared to M group).

LEfSe analysis revealed that constipation shifted the microbial landscape at the phylum level: Firmicutes was enriched in healthy controls, whereas Bacteroidetes dominated the model group (Figure 6a,b), indicating pronounced dysbiosis. At the genus level, *Parabacteroides* was markedly expanded in constipated group, while *Lachnospiraceae_NK4A136_group*, *Roseburia*, *Bifidobacterium*, *Lactobacillus*, and *Akkermansia* were depleted (Figure 6c–h; *p* < 0.001).

Intervention effects were both complementary and synergistic. The CO group selectively restored *Lactobacillus* and *Lachnospiraceae_NK4A136_group*, whereas the CE group specifically boosted *Akkermansia* and *Bifidobacterium*. The SY group combined these benefits and further elevated *Roseburia* abundance while simultaneously suppressing *Parabacteroides* (*p* < 0.05 versus model). Consequently, SY generated the most comprehensive shift toward a health-associated community profile.

This study further analyzed the content of SCFAs, the metabolic products of gut microbiota, in constipated mice. The results showed that the levels of propionic acid and butyric acid in the feces of the model group were significantly decreased (Figure 6i–k, *p* < 0.05). The intervention results revealed differential regulatory effects on SCFAs among treatment groups: the SY group exhibited significant increases in acetic acid, propionic acid, and butyric acid by 55.5%, 46.7%, and 124%, respectively (Figure 6i–k, *p* < 0.05). Both the CO and CE groups also showed significant elevations in propionic acid and butyric acid levels (44.1%, 114% and 27.6%, 118%, respectively) (Figure 6j,k, *p* < 0.05). Simultaneously, the CO group also significantly increased the acetic acid concentration by 44.1%.

In conclusion, the probiotic–prebiotic combined formulation demonstrated superior efficacy in improving gut microecology compared to individual component applications.

### 3.6. Evaluation of the Efficacy of Probiotic–Prebiotic Compound in Intestinal Immunity in Constipated Mice

**Figure 7:**
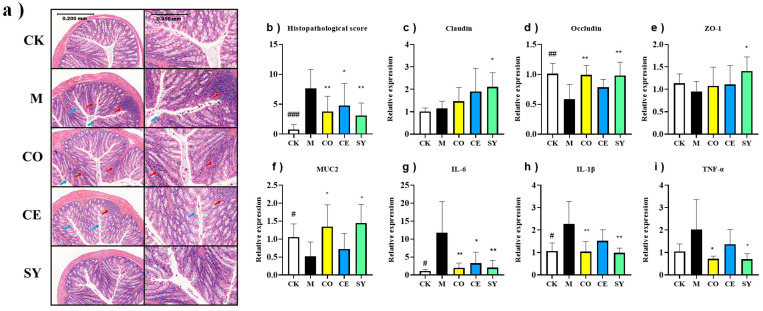
Effect of different interventions on intestinal immunity in constipated mice. (**a**) Histopathological analysis (red arrows: inflammatory infiltration; blue arrows: mucosal architectural disruption) and (**b**) histopathological score. The relative mRNA expression of (**c**) Claudin, (**d**) Occludin, (**e**) ZO-1, (**f**) MUC2, (**g**) IL-6, (**h**) IL-1β, and (**i**) TNF-α. Data represent mean ± SD. Significance levels (# *p* < 0.05, ## *p* < 0.01, ### *p* < 0.001 compared to CK group; * *p* < 0.05, ** *p* < 0.01 compared to M group).

Hematoxylin–eosin staining revealed conspicuous inflammatory infiltration (red arrows) and mucosal architectural disruption (blue arrows) in the colons of loperamide-treated mice, yielding a markedly elevated histopathological score (Figure 7a,b; *p* < 0.001). The CO and CE groups reduced the score (*p* < 0.01 and *p* < 0.05, respectively), yet residual focal leukocyte aggregates and diminished goblet-cell mucus were still evident. In contrast, the SY group restored mucosal integrity to near normal, with no significant histological lesions and a score indistinguishable from that of healthy controls (Figure 7b; *p* < 0.01 versus model).

To further investigate barrier function alterations, this study validated the effects of different treatments on intestinal mechanical barrier markers (Claudin, Occludin, ZO-1, MUC2) and immune barrier indicators (IL-6, IL-1β, TNF-α). As shown in Figure 7c–i, the model group exhibited significantly decreased colonic expression of Occludin and MUC2 (Figure 7d,f, *p* < 0.05),whereas IL-6 and IL-1β levels were markedly elevated (Figure 7g,h, *p* < 0.05), indicating constipation-induced gut barrier impairment. The intervention results revealed that compared with the model group, the SY group showed significantly upregulated expression of Claudin, Occludin, ZO-1, and MUC2 (Figure 7c–f, *p* < 0.05), with significantly downregulated expression of IL-1β, IL-6, and TNF-α (Figure 7g–i, *p* < 0.05). In contrast, the CO group only demonstrated significantly upregulated Occludin and MUC2 expression (Figure 7d, f, *p* < 0.05) and notably downregulated IL-1β, IL-6, and TNF-α levels (Figure 7g–i, *p* < 0.05). The CE group exhibited solely a significant reduction in IL-6 expression (Figure 7g, *p* < 0.05).

Comprehensive analysis demonstrated that the SY synergistically enhanced tight junction protein and mucin expression while inhibiting proinflammatory factor release, outperforming single-component interventions in restoring mechanical barrier integrity and alleviating intestinal inflammation, thereby modulating gut immunity more effectively.

### 3.7. Correlation of Constipation Phenotypes with Underlying Physiological Mechanisms

**Figure 8:**
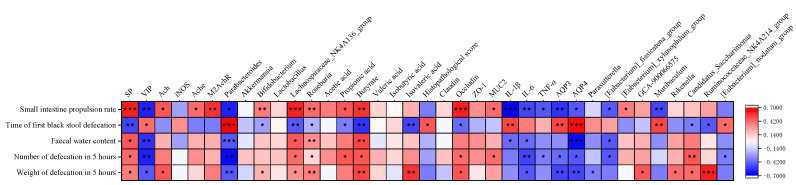
Correlation analysis between constipation phenotypic indices and mechanistic biomarkers. The colors of the cells indicate Spearman’s correlations, and asterisks in the cells represent the *p*-value adjusted by the FDR, * *p* < 0.05, ** *p* < 0.01,*** *p* < 0.001.

Pearson’s correlation analysis was performed to assess the relationship between constipation phenotype indicators and mechanistic biomarkers. As shown in Figure 8, significant positive correlations were observed between the constipation phenotype indicators (small-intestinal propulsion rate, fecal water content, 5 h fecal pellet count and weight) and the following biomarkers: SP, the relative abundances of *Lachnospiraceae_NK4A136_group* and *Roseburia*, butyrate, and Occludin. Conversely, significant negative correlations were found between these constipation phenotype indicators and the following biomarkers: VIP, AQP3, AQP4, the relative abundance of *Parabacteroides*, and IL-6. Additionally, the time to first black stool excretion showed significant negative correlations with SP, the relative abundances of *Lachnospiraceae_NK4A136_group* and *Roseburia*, butyrate, and Occludin, while significant positive correlations were observed with VIP, AQP3, AQP4, *Parabacteroides*, and IL-6.

Based on the effects of the SY group on these biomarkers, it is suggested that SY may alleviate constipation through multi-target mechanisms, including (1) modulating neurotransmitter secretion (increasing SP content and decreasing VIP content), (2) optimizing water channel protein-mediated intestinal water transport (reducing AQP3 and AQP4 content), (3) regulating gut microbiota (increasing the relative abundance of *Lachnospiraceae_NK4A136_group* and *Roseburia* and butyrate concentration while decreasing the relative abundance of *Parabacteroides*), and (4) reducing intestinal inflammation (enhancing tight junction protein Occludin expression and decreasing IL-6 levels). These combined effects contribute to improved fecal water content, small-intestinal propulsion rate, 5 h fecal pellet count and weight, and reduced time to first black stool excretion.

## 4. Discussion

Synbiotic formulations are a promising option for functional constipation. Nevertheless, most currently available synbiotic formulations are assembled empirically; the absence of quantitative matching criteria frequently blunts the synergistic potential of the bacterial–substrate combination. In the present study, we introduced GT as a rational, metric-driven screening tool. Eight oligosaccharides were evaluated for their ability to support the growth of four constipation-relieving strains (*Bifidobacterium longum* CCFM1167, *B. animalis* subsp. *lactis* CCFM1278, *B. longum* subsp. *longum* CCFM1113, *Pediococcus acidilactici* CCFM6432). For each strain, at least one substrate yielded GT values indistinguishable from glucose, allowing us to construct a GT-optimized prebiotic blend (isomaltulose, xylo-oligosaccharide, resistant dextrin, and stachyose). When this blend was combined with the four-strain probiotic, the resulting synbiotic (SY) outperformed either component alone in a loperamide-induced mouse constipation model, echoing the recent report by Yang et al. [29] showing that precise probiotic–prebiotic pairing accelerates colonic transit. It is noteworthy that certain prebiotics significantly prolonged the GT of specific strains, which may be attributed to the absence of essential carbohydrate metabolism gene clusters required for their utilization [14] or carbon-catabolite repression by constituent monosaccharides [30]. Being an in vitro monoculture assay, GT screening ignores host competition, cross-feeding, and ecological interactions; therefore, validation in continuous-culture gut models and clinical trials is essential before adopting this quantitative pipeline for precision management of functional constipation.

Beyond motility, we interrogated the neurochemical basis of constipation. FC is characterized by an imbalance between excitatory (SP, ACh) and inhibitory (VIP, NO) mediators. SP sensitizes intestinal smooth muscle to cholinergic signaling and directly promotes propagating contractions [31], whereas VIP attenuates motility by hyperpolarizing muscle layers and stimulating fluid secretion [32]. ACh binds to muscarinic receptors—especially the M2 subtype—to initiate peristalsis, and its action is terminated by AChE in a feedback-regulated manner [33,34]. We found that SY simultaneously elevated colonic SP, ACh, AChE, and M2AChR transcripts while suppressing VIP and iNOS activity, an effect more comprehensive than that achieved by either monotherapy. Correlation analysis confirmed that SP levels paralleled transit rate and stool hydration, whereas VIP correlated inversely with these indices, underscoring the therapeutic value of a bidirectional neurotransmitter reset. These findings align with Jiang et al. [35], who also demonstrated that combined probiotics and prebiotics improve constipation by modulating enteric neuropeptides.

SP treatment enhanced striatal cholinergic interneuron neuronal activity and ACh release in the striatum through neurokinin 1 receptor activation [36]. In the present study, both prebiotic and SY interventions upregulated colonic levels of SP and ACh, suggesting that they may enhance intestinal motility through SP-ACh signaling pathways. Simultaneously, the probiotic and SY treatments downregulated intestinal VIP levels while increasing MUC2 mRNA expression. Since Song et al. demonstrated that VIP inhibits mast cell activation and mucus secretion [37], our results imply that these interventions may reinforce the intestinal mucus barrier by modulating the VIP–mast cell–MUC2 axis. Transcript-level changes do not necessarily correspond to functional protein activity. Further validation using protein-level assays, such as Western blotting, is required to confirm alterations in the relevant signaling pathways mediated by these neurochemicals.

Excessive colonic water absorption is another cardinal feature of FC. Aquaporins 3 and 4 are the principal trans-cellular water transporters in the murine and human colon; their over-expression dehydrates luminal content and hardens stools [38]. In our model, AQP3/AQP4 mRNA abundance rose markedly, but SY downregulated both channels and increased fecal water content by 78%. Guo et al. [25] reported a similar AQP-lowering effect following synbiotic intervention, suggesting that microbiota-mediated signaling—possibly via SCFA-activated G-protein-coupled receptors—restrains epithelial water re-uptake. Notably, in our study, all three intervention groups exhibited significant upregulation of fecal SCFAs. This suggested that prebiotic, probiotic, and SY treatments may increase fecal hydration by enhancing SCFA production and subsequently downregulating AQP3/AQP4 expression, thereby contributing to the alleviation of constipation symptoms.

Collectively, the GT-guided synbiotic exerts multi-target remediation: it restores enteric neurotransmitter balance, limits aquaporin-driven water removal, enriches SCFA-producing commensals, and reinforces barrier integrity, thereby providing a rational, translational framework for precision microecological therapy of functional constipation.

The pathogenesis of functional constipation is intimately linked to dysbiosis. A skewed microbiome compromises the mechanical barrier and erodes the mucus layer, permitting luminal antigens to translocate and trigger a pro-inflammatory cascade that aggravates mucosal injury [39,40,41,42]. Conversely, beneficial taxa that generate short-chain fatty acids—especially butyrate—reinforce tight-junction integrity and exert anti-inflammatory effects, thereby establishing a protective feedback loop [39,40,41,42]. Probiotics and prebiotics are therefore believed to relieve constipation primarily by rebalancing microbial composition and restoring metabolite homeostasis [11].

In the present, study the GT-optimized synbiotic selectively enriched *Lachnospiraceae_NK4A136_group* and *Roseburia* while suppressing *Parabacteroides*, a pattern that coincided with a 124% surge in colonic butyrate and parallel improvements in barrier markers and motility indices. These findings corroborate previous observations: Yang et al. [29] identified *Parabacteroides* as a conditional pathobiont whose expansion compromises epithelial integrity and fuels inflammation; Zhang [43] demonstrated that probiotic-induced expansion of *Roseburia* elevates fecal butyrate, accelerates colonic migration, and ameliorates mucosal dysfunction; and Jiang [44] reported a positive correlation between *Lachnospiraceae_NK4A136_group* abundance and both Occludin and SP levels, together with an inverse correlation with IL-6. Collectively, SY orchestrates a microbiota–metabolite–host axis that re-establishes epithelial integrity and neuromuscular signaling. Intra-individual variability in the gut microbiota represents a significant component of total compositional variance [45]. The enrichment or suppression of specific taxa observed in our study may vary considerably among individuals. Future investigations should incorporate longitudinal fecal sampling to control for intra-individual variability and validate the consistency of microbial shifts. Ultimately, validation in diverse human cohorts is essential to confirm the generalizability of our GT-optimized synbiotic approach for functional constipation.

By adopting generation time as a quantitative design criterion, we provide a proof of concept for metabolism-driven, precision synbiotic construction. The resultant formulation simultaneously normalizes enteric neurotransmission, aquaporin-mediated water flux, keystone taxa abundance, barrier integrity, and inflammatory tone—outperforming either probiotic or prebiotic alone. The strategy offers a promising route to rationally designed consortia, although the current GT assay remains an in vitro single-substrate model. The research is currently at the preclinical stage, and future work should integrate continuous-culture gut simulators and in vivo validation experiments to refine the dose–response evaluation system. Before considering any clinical translation, rigorous human trials are essential to verify its safety and efficacy.

## 5. Conclusions

**Figure 9:**
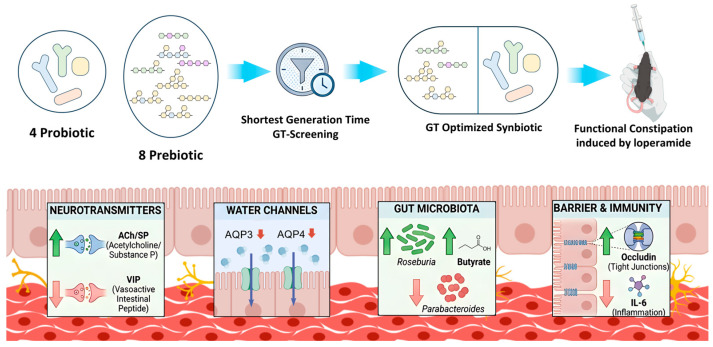
Mechanism of the GT-optimized synbiotic in alleviating functional constipation. Figure 9 was created with BioGDP.com [46].

This study provides proof-of-concept evidence that GT may serve as a quantitative criterion for synbiotic design. By systematically profiling the substrate preferences of functionally characterized strains, we identified optimal prebiotic counterparts. The resulting GT-optimized synbiotic markedly outperformed either probiotics CO or prebiotics CE alone in a loperamide-induced mouse model, warranting validation in human studies. Mechanistically, SY appears to operate through multiple pathways, including modulation of neurotransmitters, aquaporins, microbiota, and inflammation (Figure 9). This strategy offers a promising framework for rational synbiotic design, though further validation in continuous culture systems and clinical trials is required before precision application in functional constipation. 

## Figures and Tables

**Table 1:** Information on experimental strains.

Strain	Source
*Bifidobacterium bifidum* CCFM1167	Female feces
*Bifidobacterium animalis* subsp. *lactis* CCFM1278	Feces of elderly men
*Bifidobacterium longum* subsp. *longum* CCFM1113	Male feces
*Pediococcus acidilactici* CCFM6432	Feces of men

**Table 2:** Information on experimental groups in the animal study of functional constipation.

Group	Substances Administered by Gavage	Concentration	Volume
CK	Sterile physiological saline	-	0.2 mL
M	Loperamide hydrochloride	10 mg/(kg·bw)	0.2 mL
CO	10^9^ CFU/mL *B. bifidum* CCFM1167 + 10^9^ CFU/mL *B. animalis* CCFM1278 + 10^9^ CFU/mL *B. longum* CCFM1113 + 10^9^ CFU/mL *P. acidilactici* CCFM6432	1 × 10^9^ CFU/mL	0.2 mL
CE	0.02 g/mL isomaltulose + 0.04 g/mL xylo-oligosaccharide + 0.1 g/mL resistant dextrin + 0.06 g/mL stachyose	0.22 g/mL	0.2 mL
CY	CO + CE	1 × 10^9^ CFU/mL + 0.22 g/mL	0.2 mL

**Table 3:** Primer sequences used in this study.

Primer Name	Primer Sequences
Gapdh-F	TGTGGATGGCCCCTCTGGAA
Gapdh-R	TGACCTTGCCCACAGCCTTG
Occludin-F	TTGAAAGTCCACCTCCTTACAGA
Occludin-R	CCGGATAAAAAGAGTACGCTGG
MUC2-1-F	GCCCACCTCACAAGCAGTAT
MUC2-1-R	GTCATAGCCAGGGGCAAACT
IL-1β-F	GAAATGCCACCTTTTGACAGTG
IL-1β-R	TGGATGCTCTCATCAGGACAG
IL-6-F	CTGCAAGAGACTTCCATCCAG
IL-6-R	AGTGGTATAGACAGGTCTGTTGG
AQP4-F	GCAGACAAGGTGCAACGTGGTT
AQP4-R	GGCGGAAGGCAAAGCAGTATGG
AQP3-F	CCTGGTGGTCCTGGTCATTGG
AQP3-R	GCGGTGAAGAGGCGAGGTC
M2AchR-F	TAGTGGGATCGTCAGGTCAGAATG
M2AchR-R	CAGGCTGCTTGGTCATCTTCAC
AChE-F	AGCCTGAACCTGAAGCCCTTAG
AChE-R	CCGCCTCGTCCAGAGTATCG

## Data Availability

The original contributions presented in this study are included in the article. Further inquiries can be directed to the corresponding authors.
